# P-609. Epidemiology and Outcomes of Nocardia Colonization

**DOI:** 10.1093/ofid/ofaf695.822

**Published:** 2026-01-11

**Authors:** Julia Patricia Bunal, Maria Vega Brizneda, Anisha Misra, Susan Harrington, Cyndee Miranda, Eric Cober, Zachary Yetmar

**Affiliations:** Cleveland Clinic Foundation, Cleveland, Ohio; Cleveland Clinic, Cleveland, OH; Cleveland Clinic Foundation, Cleveland, Ohio; Cleveland Clinic, Cleveland, OH; Cleveland Clinic, Cleveland, OH; Cleveland Clinic Foundation, Cleveland, Ohio; Cleveland Clinic, Cleveland, OH

## Abstract

**Background:**

Nocardia species are known to cause both respiratory colonization and pulmonary nocardiosis, with the latter leading to significant morbidity and mortality. There is limited data showing a distinction between colonization and active disease, as well as disease progression and optimal management strategies. This study compares individuals with respiratory Nocardia colonization to those diagnosed with pulmonary nocardiosis, with a focus on baseline characteristics, survival outcomes, and risk factors associated with disease progression.

**Table 1.**
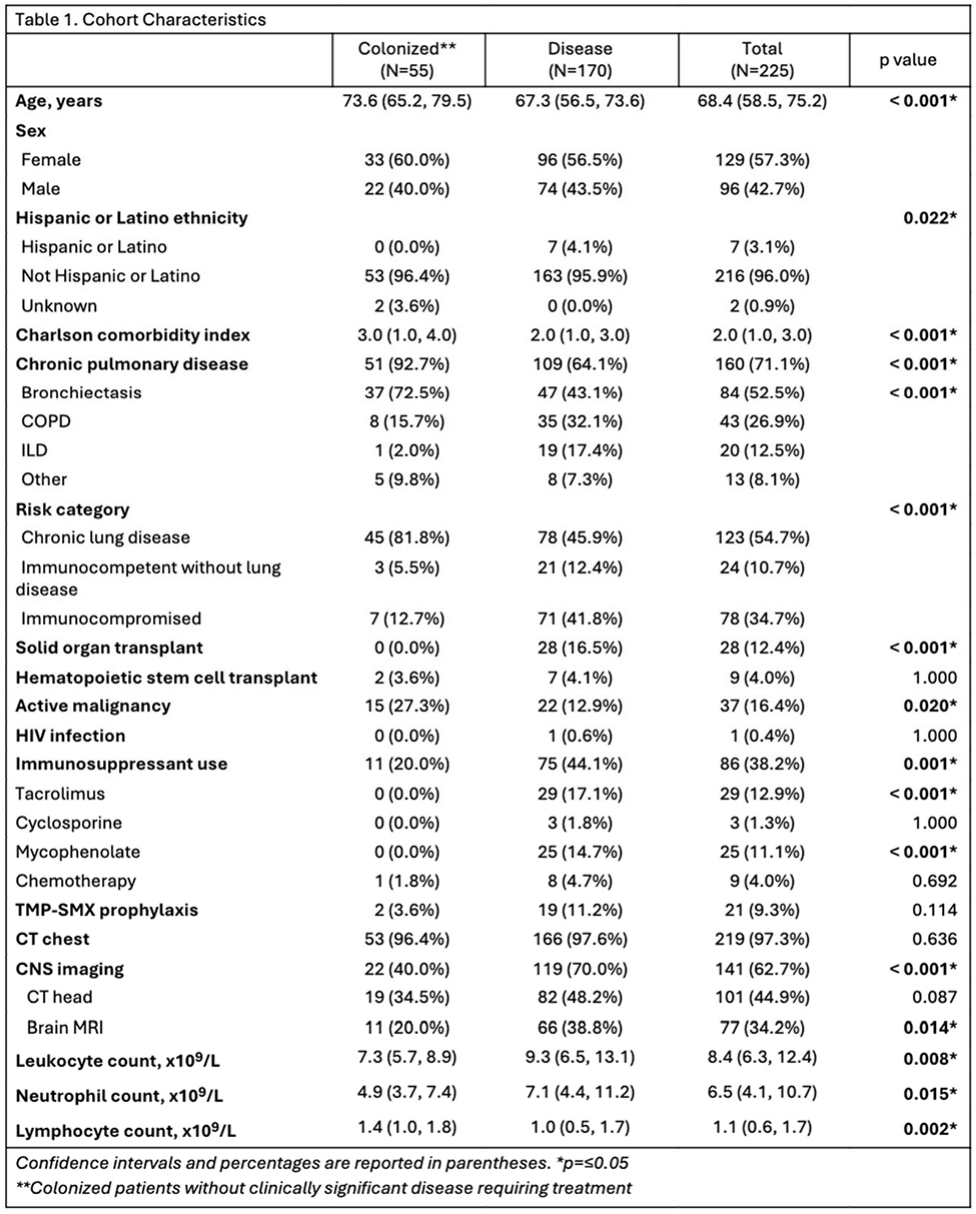
Cohort Characteristics Epidemiology and Outcomes of Nocardia Colonization

**Methods:**

We analyzed 225 patients with Nocardia species isolated in respiratory cultures from 2000 to 2023 at a tertiary hospital in Cleveland, Ohio. Nocardiosis was defined as Nocardia growth in culture with accompanying signs, symptoms, and radiologic findings consistent with active infection. Nocardia colonization was the isolation of Nocardia on cultures without clinical disease. We conducted a retrospective cohort study comparing two groups: individuals with respiratory Nocardia colonization and those with pulmonary nocardiosis. Factors associated with colonization were assessed by multivariable logistic regression.

**Results:**

Fifty-five (24.4%) patients with Nocardia colonization and 170 patients (75.6%) with nocardiosis were included (Table 1). Immunocompetent patients with chronic lung disease (adjusted OR 7.73, CI 3.37-19.95, p≤0.001) and patients with a higher Charlson comorbidity index (OR 1.52 per point, 95% CI 1.27-1.85, p≤0.001) had an increased likelihood of Nocardia colonization. Lung cavitation was associated with nocardiosis (adjusted OR 0.21, 95% CI 0.05-0.68, p=0.020), while identification of N. farcinica was not statistically significant (adjusted OR 0.28, 95% CI 0.04-1.05, p=0.102). Patients with colonization had a lower, though non-significant one-year mortality rate (7.3% versus 17.1%, log-rank p=0.061).

**Conclusion:**

These findings delineate important differences in patient characteristics and survival outcomes between respiratory Nocardia colonization and pulmonary nocardiosis, highlighting the need for prospective studies to identify colonized patients who are at risk for disease progression.

**Disclosures:**

Susan Harrington, PhD, Bruker Daltonics, Inc: Grant/Research Support

